# Constipation might be associated with risk of allergic rhinitis: A nationwide population-based cohort study

**DOI:** 10.1371/journal.pone.0239723

**Published:** 2020-10-02

**Authors:** Meng-Che Wu, Ming-Shiou Jan, Jeng-Yuan Chiou, Yu-Hsun Wang, James Cheng-Chung Wei

**Affiliations:** 1 Institute of Medicine, Chung Shan Medical University, Taichung, Taiwan; 2 Division of Gastroenterology, Children’s Medical Center, Taichung Veterans General Hospital, Taichung, Taiwan; 3 Institute of Biochemistry, Microbiology and Immunology, Chung Shan Medical University, Taichung, Taiwan; 4 Immunology Research Center, Chung Shan Medical University, Taichung, Taiwan; 5 Division of Allergy, Immunology and Rheumatology, Chung Shan Medical University Hospital, Taichung, Taiwan; 6 School of Health Policy and Management, Chung Shan Medical University, Taichung, Taiwan; 7 Department of Medical Research, Chung Shan Medical University Hospital, Taichung, Taiwan; 8 Graduate Institute of Integrated Medicine, China Medical University, Taichung, Taiwan; Ohio State University, UNITED STATES

## Abstract

**Background:**

Allergic rhinitis (AR) is a burdensome respiratory disorder whose etiology and pathophysiology remain controversial and most likely multifactorial. Accumulated evidence indicates that gut dysbiosis contributes to AR via the gut-airway axis. Constipation could result in alteration of the intestinal microflora. The clinical impact of constipation on AR has not been studied. We aimed to evaluate the risk of AR in constipated patients using a nationwide longitudinal population-based cohort.

**Methods:**

We identified 57786 patients with constipation and 57786 matched controls between 1999 and 2013 from the Longitudinal Health Insurance Database, which is a subset of Taiwanese National Health Insurance Research Database. Propensity score analysis was used for matching age, sex, comorbidities, and medications at a ratio of 1:1. Multiple Cox regression and subgroup analyses were used to estimate the adjusted hazard ratio of AR.

**Results:**

The incidence of AR was 32.2 per 1,000 person-years in constipated patients, which was twice that of non-constipated patients. After adjustment for patients’ age, gender, comorbidities, and medications, patients with constipation had a 2.3-fold risk of AR compared to those without constipation (adjusted hazard ratio [aHR]: 2.30; 95% CI, 2.23–2.37). In subgroup analyses, patients aged 20–39 years had a 2.24-fold higher risk of AR in the constipation cohort (aHR; 95% CI, 2.12–2.36). Patients aged <20, 40–64, and ≥65 years had a 2.09, 2.05, and 2.07-fold risk of AR in the constipation cohort, respectively (aHR; 95% CI, 1.98–2.20, 1.94–2.18, and 1.92–2.23). Also, patients with constipation had a higher likelihood of AR, regardless of sex, and with or without comorbidities including hyperlipidemia, hypertension, chronic kidney disease, chronic liver disease, diabetes, chronic obstructive pulmonary disease, rheumatoid arthritis, dyspepsia, irritable bowel syndrome, and anxiety.

**Conclusion:**

Constipation might be associated with an increased risk of incidental AR. It seems that physicians should keep a higher index of suspicion for AR in people with constipation. The patency issue of gut could not be ignored in patients with AR.

## Introduction

Allergic rhinitis (AR) is characterized by paroxysmal sneezing, nasal stuffiness, postnasal drainage, rhinorrhea, and itchy nose. During the past few decades, the prevalence of AR has increased dramatically around the globe [1]. It affects approximately 10 to 30% of adults and children in industrialized countries [2, 3], and is associated with economic loss and significant morbidity. AR is a burdensome respiratory disorder, but its etiology and pathophysiology have not yet been fully elucidated. Constipation is one of the most common, multifactorial gastrointestinal disorders and its median prevalence worldwide ranges from 8.2% to 32.9% [4]. Complications of constipation include anal fissures, urine or fecal incontinence, hemorrhoids, and rectal prolapse, which often increase the frequency of outpatient visits or hospitalizations, thereby increasing the cost of health insurance. It is also becoming increasingly frequent and is considered a major health issue that has a significant negative impact on the quality of life (QoL) [5]. The deleterious effect of constipation on QoL has been shown to be comparable or even more severe than other chronic conditions like inflammatory bowel disease, diabetes, rheumatoid arthritis, and hemodialysis [5].

A shred of studies have suggested that atopic disease might be linked to constipation [6–8]. Studies have reported an indirect indication of a concurrence of constipation and atopy by demonstrating a high prevalence of coexistent allergic manifestations in constipated children investigated for cow milk allergy [9, 10]. An epidemiologic survey by Tokunaga et al. showed that constipation was a relevant factor for AR development (adjusted odds ratio of 1.17) among 21802 high school students [11]. Jones et al. stated that the overlap of atopy in functional gastrointestinal disorders patients, and the risk of rhinitis in 342 constipated patients was 1.66 times higher than controls [8]. Previous research also demonstrated that prolonged stool stasis in the colon had a significant impact on the intestinal ecosystem, which could affect a variety of bowel functions, including motility and mucosal immunity [12–16]. Constipation is currently considered to be a causative factor in intestinal dysbiosis [13, 17]. For instances, a study by Khalif et al. in Ireland reported a decreased abundance in Bifidobacteria and Lactobacillus [17]. A clinical trial by Kim et al. in Korea showed a decreased abundance of Bacteroides and Bifidobacterium species, when compared to the non-constipated controls [18]. Recent research using 16S rRNA gene-based microbiome analysis documented dysbiosis of gut microbiota in constipated patients [13, 14, 19, 20]. Hence, in addition to laxatives, manipulation of the gut microbiome has increasingly been seen as a novel target for therapeutic possibilities for constipation [2, 15, 20, 21]. Likewise, differences in the composition of gut microbiome have been demonstrated when comparing AR subjects and healthy controls [22–24]. Moreover, accumulated evidence shows that intestinal dysbiosis is associated with an increased risk of AR via gut-airway axis [2, 20, 25–27]. However, little is known about whether constipation could influence AR. Data obtained from a real-world large national longitudinal database have never been utilized for investigating this relationship. We thus hypothesized that constipation could impact the risk of AR and evaluated this hypothesis by analyzing a nationwide population-based retrospective cohort from the Taiwanese National Health Insurance Research Database (NHIRD).

## Materials and methods

### Ethics approval and consent to participate

This study was approved by the Institutional Review Board of Chung Shan Medical University Hospital (Approval number CS15134) in Taiwan. The requirement for written consent from study subjects was waived by the Institutional Review Board, as the LHID consists of de-identified secondary data.

### Data source and study population

This retrospective cohort study was conducted by using data from the National Health Insurance Research Database (NHIRD), a database covering 99% of Taiwan’s population of 23 million beneficiaries. This database includes all insurance claims data, such as outpatient visits, emergency visits, and hospitalizations. One million subjects (Longitudinal Health Insurance Database, LHID) [28–30] were randomly sampled from the 23 million beneficiaries, providing data between 1999 and 2013. This sampled database was de-identified in accordance with privacy protocols. The Institutional Review Board of Chung Shan Medical University Hospital (Approval number CS15134) has approved this study.

**Fig 1:**
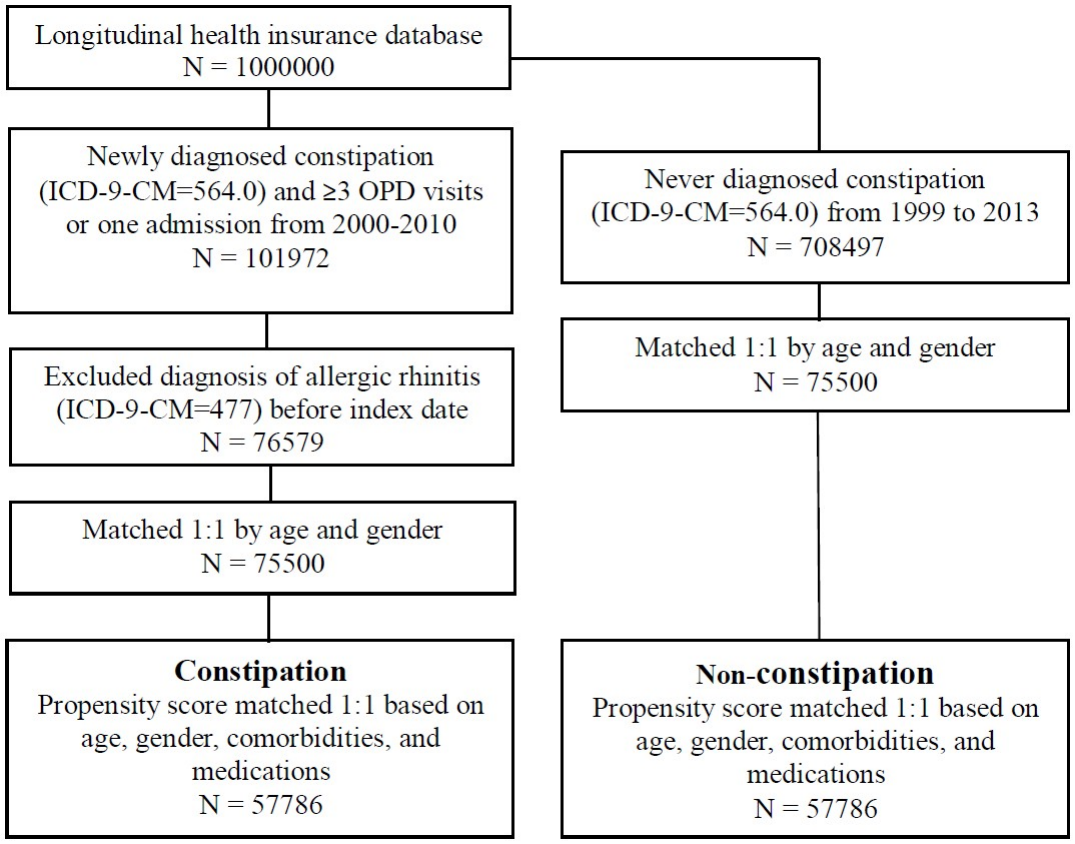
Flowchart of study.

The study population comprised patients with newly diagnosed constipation (ICD-9-CM codes = 564.0) from 2000 to 2010. To ensure the accuracy of diagnoses, only patients with at least three outpatient visits or one hospitalization were included. The index date of this cohort was set as the date of first diagnosis of constipation. In order to ensure that only new-onset subjects were enrolled, patients diagnosed with allergic rhinitis (ICD-9-CM = 477) before the index date were excluded. The non-constipation group was composed of patients who had never been diagnosed with constipation (ICD-9-CM = 564.0) for the period 1999 to 2013. The index date for the non-constipation group was determined according to the respective matched cases. The flowchart of enrolment is depicted in Fig 1.

The outcome variable was defined as a diagnosis of allergic rhinitis (ICD-9-CM = 477) with at least three outpatient visits or one hospitalization. This study was followed up till the occurrence of AR, 31 December 2013, or until their records were censored for death, emigration, or discontinuation of enrolment in the National Health Insurance system.

### Covariates and matching

The baseline characteristics were age, gender, related comorbidities including hyperlipidemia (ICD-9-CM = 272.0–272.4), hypertension (ICD-9-CM = 401–405), chronic kidney disease (ICD-9-CM = 585), chronic liver disease (ICD-9-CM = 571), diabetes (ICD-9-CM = 250), chronic obstructive pulmonary disease (COPD) (ICD-9-CM = 491, 492, 496), autoimmune diseases such as systemic lupus erythematosus (SLE) (ICD-9-CM = 710.0), rheumatoid arthritis (RA) (ICD-9-CM = 714.0), Sjogren’s syndrome (ICD-9-CM = 710.2), ankylosing spondylitis (AS) (ICD-9-CM = 720.0), and the diseases predisposing constipation [31], including dyspepsia (ICD-9-CM = 536.8), gastroesophageal reflux disease(GERD) (ICD-9-CM = 530.11, 530.8x), irritable bowel syndrome (IBS) (ICD-9-CM = 564.1), gastrointestinal tract cancers (ICD-9-CM = 150–159), colonic polyp (ICD-9-CM = V12.72, 211.3 and 211.4), anxiety (ICD-9-CM = 300.0), depression (ICD-9-CM = 296.2, 296.3, 300.4 and 311), hypothyroidism (ICD-9-CM = 243, 244), Parkinson’s disease (ICD-9-CM = 322), multiple sclerosis (ICD-9-CM = 340), spinal cord injury (ICD-9-CM = 806, 952). The comorbidities were defined as occurring within one year prior to the index date and with at least three outpatient visits or one hospitalization. In addition, medications containing corticosteroids, antihistamines, non-steroidal anti-inflammatory drugs, calcium channel blockers, diuretics, opioids, antidepressants, serotonin antagonists, anticonvulsants, antispasmodic, iron supplement, calcium supplement during the study period were included and defined as usage for ≥30 days.

Propensity-score matching was performed to match the two groups based on age, gender, hypertension, hyperlipidemia, chronic liver disease, diabetes, chronic kidney disease, COPD, SLE, RA, Sjogren’s syndrome, AS, dyspepsia, GERD, IBS, gastrointestinal tract cancers, colonic polyp, anxiety, depression, hypothyroidism, Parkinson’s disease, multiple sclerosis, spinal cord injury, and various medications. The propensity score was the probability estimated by logistic regression, with the binary variable being whether or not patients had constipation, i.e., constipation vs. non-constipation groups. Propensity-score matching was performed in order to balance the heterogeneity of the two groups [32].

### Statistical analysis

The constipation group and non-constipation group were compared by Chi-square test or Fisher’s exact test for categorical variables and Student’s t- test for continuous variables. Moreover, we also used absolute standardized differences (ASD) to perform the difference between the two groups. When the absolute standardized difference was less than 0.1, the characteristics of both groups were considered to be similar [33]. Kaplan-Meier analysis was used to calculate the cumulative incidence of AR from the index date and log-rank test was used to test the significance. Cox proportional hazard model was used to estimate the hazard ratio of AR between the constipation group and non-constipation group. SPSS version 18.0 (SPSS Inc., Chicago, IL, USA) was used for all statistical analyses.

## Results

**Fig 2:**
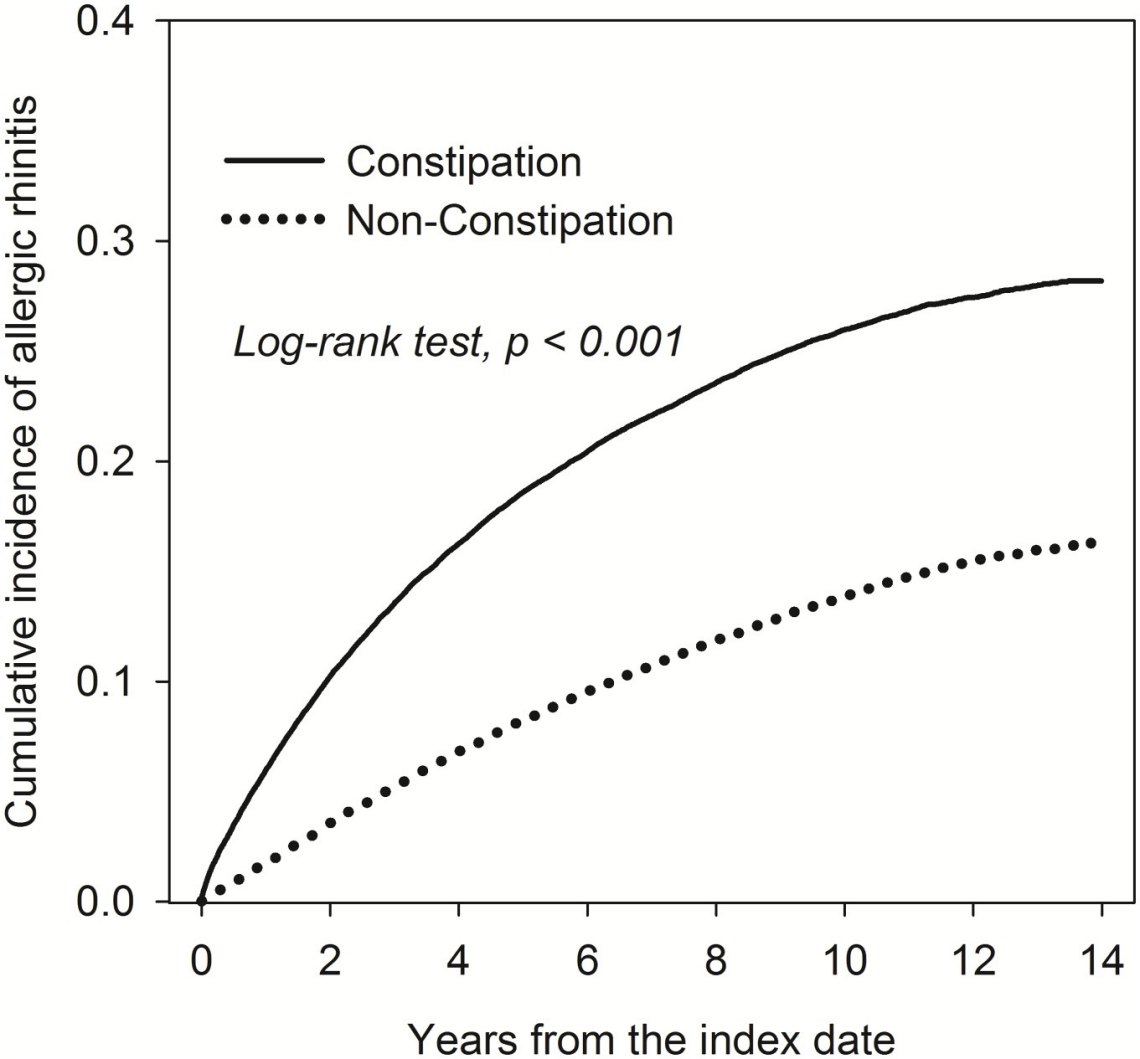
Kaplan–Meier curves of the cumulative probability of AR in the study groups.

We identified 57786 patients with constipation and 57786 matched controls between 1999 and 2013 from the LHID, a subset of Taiwanese NHIRD. Table 1 shows the demographic characteristics of the patients. The constipation and non-constipation groups were similar in age and gender distribution; however, women had twice the incidence of constipation compared with men. Also, there were no statistically significant differences between the constipation cohort and the non-constipation cohort after propensity score matching. Table 2 displays the incidence density and risk factors for AR. The incidence of AR was 32.2 per 1,000 person-years in constipation patients, which was higher than the rate of 14.8 per 1,000 person-years found in non-constipation patients. After adjustment, patients with constipation had a significantly higher risk of AR than those without constipation (aHR, 2.30; 95% CI, 2.23–2.37; P < 0.001), and older age groups were associated with a lower risk of developing AR when compared with the <20 years age group. Compared with women, men had a higher risk of AR (aHR, 1.07; 95% CI, 1.04–1.10; P < 0.001). In terms of comorbidities, patients with hypertension(aHR, 1.61; 95% CI, 1.52–1.71; P < 0.001), hyperlipidemia (aHR, 1.23; 95% CI, 1.13–1.34; P < 0.001), chronic liver disease (aHR, 1.44; 95% CI, 1.31–1.58; P < 0.001), COPD (aHR, 2.02; 95% CI, 1.84–2.21; P < 0.001), RA (aHR, 1.58; 95% CI, 1.18–2.12; P = 0.002), AS (aHR, 1.81; 95% CI, 1.07–3.06; P = 0.027), SLE (aHR, 1.88; 95% CI, 1.25–2.83; P = 0.002), Sjogren’s syndrome(aHR, 2.45; 95% CI, 1.68–3.58; P< 0.001), dyspepsia (aHR, 1.34; 95% CI, 1.18–1.52; P < 0.001), IBS (aHR, 1.45; 95% CI, 1.21–1.73; P < 0.001), anxiety (aHR, 1.69; 95% CI, 1.52–1.88; P < 0.001) and depression (aHR, 1.41; 95% CI, 1.22–1.63; P < 0.001) were at higher risk of AR. In Table 3, subgroup analyses were performed to assess the relationship between constipation and AR based on demographic characteristics. In patients aged 20–39 years, compared with the non-constipation cohort, there was a 2.24-fold higher risk of AR in the constipation cohort (aHR; 95% CI, 2.12–2.36; P < 0.001). Patients aged <20, 40–64, and ≥65 years had a 2.09, 2.05, and 2.07-fold risk of AR in the constipation cohort (aHR; 95% CI, 1.98–2.20, 1.94–2.18 and 1.92–2.23; P < 0.001), respectively. Among women, compared with patients without constipation, there was a 2.13-fold higher risk of AR in patients with constipation (aHR; 95% CI, 2.05–2.20; P < 0.001). Among men, there was 2.02-fold higher risk of AR in patients with constipation (aHR; 95% CI, 1.92–2.13; P < 0.001). Furthermore, patients with constipation had a significantly higher likelihood of AR, regardless of sex, and with or without comorbidities including hyperlipidemia, hypertension, chronic kidney disease, chronic liver disease, diabetes, COPD, RA, dyspepsia, IBS, and anxiety. The interaction effect was to compare the hazard ratios between different subgroups. In subgroup of non-hypertension, non-hyperlipidemia, non-chronic liver disease, and non-depression, constipation group had significant higher risk of allergic rhinitis. The Kaplan–Meier curves are shown in Fig 2. The cumulative incidence of AR was significantly higher in constipated patients than non-constipated patients, and the log-rank test for the comparison of cumulative incidence curves resulted in a P-value of <0.001.

**Table 1:** Demographic characteristics of constipation group and non-constipation group.

	Before propensity score matched						After propensity score matched					
	Constipation		Non-constipation				Constipation		Non-constipation			
	(N = 75500)		(N = 75500)				(N = 57786)		(N = 57786)			
	n	%	n	%	ASD	p-value	n	%	N	%	ASD	p-value
Age					<0.001	1					0.026	0.002
<20	11001	14.6	11001	14.6			9722	16.8	10059	17.4		
20–39	20395	27.0	20395	27.0			16677	28.9	16274	28.2		
40–64	22337	29.6	22337	29.6			16194	28.0	16484	28.5		
≥65	21767	28.8	21767	28.8			15193	26.3	14969	25.9		
Mean ± SD	46.4 ± 23.6		46.4 ± 23.6		<0.001	1	44.4 ± 24		44 ± 23.8		0.018	0.002
Gender					<0.001	1					0.003	0.569
Female	49243	65.2	49243	65.2			38615	66.8	38706	67.0		
Male	26257	34.8	26257	34.8			19171	33.2	19080	33.0		
Hypertension	15253	20.2	10458	13.9	0.170	<0.001	9082	15.7	9043	15.6	0.002	0.752
Hyperlipidemia	3938	5.2	2615	3.5	0.086	<0.001	2275	3.9	2256	3.9	0.002	0.773
Chronic liver disease	3058	4.1	1497	2.0	0.121	<0.001	1465	2.5	1407	2.4	0.006	0.273
Chronic kidney disease	883	1.2	493	0.7	0.054	<0.001	435	0.8	415	0.7	0.004	0.491
Diabetes	7808	10.3	4387	5.8	0.167	<0.001	4085	7.1	4020	7.0	0.004	0.454
COPD	3128	4.1	1573	2.1	0.119	<0.001	1491	2.6	1484	2.6	0.001	0.897
Rheumatoid arthritis	281	0.4	165	0.2	0.028	<0.001	148	0.3	143	0.2	0.002	0.769
Ankylosing spondylitis	92	0.1	34	0.05	0.027	<0.001	37	0.1	34	0.1	0.002	0.722
SLE	75	0.1	60	0.1	0.007	0.197	52	0.1	45	0.1	0.004	0.477
Sjogren’s syndrome	124	0.2	65	0.1	0.022	<0.001	55	0.1	61	0.1	0.003	0.577
Dyspepsia	1867	2.5	567	0.8	0.137	<0.001	615	1.1	566	1.0	0.008	0.152
GERD	528	0.7	127	0.2	0.081	<0.001	145	0.3	127	0.2	0.006	0.275
Irritable bowel syndrome	955	1.3	281	0.4	0.099	<0.001	321	0.6	281	0.5	0.010	0.102
Gastrointestinal tract cancer	905	1.2	502	0.7	0.056	<0.001	489	0.8	460	0.8	0.006	0.345
Colonic polyp	170	0.2	53	0.1	0.040	<0.001	66	0.1	53	0.1	0.007	0.233
Anxiety	2566	3.4	956	1.3	0.142	<0.001	984	1.7	931	1.6	0.007	0.222
Depression	1988	2.6	534	0.7	0.151	<0.001	584	1.0	527	0.9	0.010	0.086
Hypothyroidism	213	0.3	117	0.2	0.027	<0.001	122	0.2	110	0.2	0.005	0.430
Parkinson’s disease	879	1.2	250	0.3	0.097	<0.001	247	0.4	250	0.4	0.001	0.893
Multiple sclerosis	10	0.01	2	0.003	0.012	0.021	1	0.002	2	0.003	0.003	0.999
Spinal cord injury	267	0.4	48	0.1	0.064	<0.001	55	0.1	48	0.1	0.004	0.490
Corticosteroids	20240	26.8	12612	16.7	0.247	<0.001	11729	20.3	11673	20.2	0.002	0.682
Antihistamines	51295	67.9	38014	50.3	0.364	<0.001	35663	61.7	35677	61.7	0.000	0.932
NSAIDs	55459	73.5	43706	57.9	0.332	<0.001	39317	68.0	39457	68.3	0.005	0.377
Calcium channel blockers	21533	28.5	16949	22.4	0.140	<0.001	13656	23.6	13556	23.5	0.004	0.488
Diuretics	18886	25.0	12421	16.5	0.212	<0.001	10842	18.8	10868	18.8	0.001	0.845
Opioids	9228	12.2	4973	6.6	0.194	<0.001	4657	8.1	4583	7.9	0.005	0.422
Antidepressants	14216	18.8	5566	7.4	0.345	<0.001	5698	9.9	5455	9.4	0.014	0.015
Serotonin (5HT3) antagonists	772	1.0	472	0.6	0.044	<0.001	428	0.7	444	0.8	0.003	0.587
Anticonvulsants	11968	15.9	4833	6.4	0.304	<0.001	4910	8.5	4681	8.1	0.014	0.015
Antispasmodic	19669	26.1	11406	15.1	0.273	<0.001	10934	18.9	10894	18.9	0.002	0.764
Iron supplement	4229	5.6	2365	3.1	0.121	<0.001	2212	3.8	2176	3.8	0.003	0.580
Calcium supplement	7654	10.1	4677	6.2	0.144	<0.001	4186	7.2	4153	7.2	0.002	0.708

ASD: Absolute standardized differences.

COPD: Chronic obstructive pulmonary disease.

SLE: Systemic lupus erythematosus.

GERD: Gastroesophageal reflux disease.

NSAIDs: Non-steroidal anti-inflammatory drugs.

**Table 2:** Multiple Cox proportional hazard regression for the estimation of adjusted hazard ratios for AR.

	No. of allergic rhinitis	Observed Person-Years	ID	Crude HR	95% C.I.	p value	Adjusted HR^†^	95% C.I.	p value
Group									
Non-constipation	7104	480068	14.8	1			1		
Constipation	13076	405708	32.2	2.09	2.03–2.15	<0.001	2.30	2.23–2.37	<0.001
Age						<0.001			<0.001
<20	6097	163913	37.2	1			1		
20–39	5952	270959	22.0	0.57	0.55–0.59	<0.001	0.58	0.56–0.6	<0.001
40–64	4924	262465	18.8	0.48	0.47–0.5	<0.001	0.62	0.6–0.65	<0.001
≥65	3207	188439	17.0	0.41	0.4–0.43	<0.001	0.65	0.62–0.69	<0.001
Gender									
Female	13787	617078	22.3	1			1		
Male	6393	268699	23.8	1.04	1.01–1.07	0.022	1.07	1.04–1.1	<0.001
Hypertension	2219	113499	19.6	0.78	0.75–0.82	<0.001	1.61	1.52–1.71	<0.001
Hyperlipidemia	642	28879	22.2	0.91	0.84–0.98	0.014	1.23	1.13–1.34	<0.001
Chronic liver disease	466	17869	26.1	1.10	1–1.2	0.048	1.44	1.31–1.58	<0.001
Chronic kidney disease	69	3696	18.7	0.71	0.56–0.9	0.004	0.95	0.75–1.21	0.678
Diabetes	822	47744	17.2	0.69	0.64–0.73	<0.001	0.86	0.8–0.93	<0.001
COPD	528	13425	39.3	1.55	1.42–1.69	<0.001	2.02	1.84–2.21	<0.001
Rheumatoid arthritis	46	1887	24.4	1.04	0.78–1.39	0.783	1.58	1.18–2.12	0.002
Ankylosing spondylitis	14	475	29.5	1.24	0.74–2.1	0.412	1.81	1.07–3.06	0.027
SLE	24	649	37.0	1.55	1.04–2.32	0.031	1.88	1.25–2.83	0.002
Sjogren’s syndrome	28	653	42.9	1.71	1.18–2.48	0.005	2.45	1.68–3.58	<0.001
Dyspepsia	244	7542	32.4	1.37	1.21–1.55	<0.001	1.34	1.18–1.52	<0.001
GERD	24	1283	18.7	0.68	0.45–1.01	0.056	0.59	0.4–0.88	0.010
Irritable bowel syndrome	121	3789	31.9	1.35	1.13–1.61	0.001	1.45	1.21–1.73	<0.001
Gastrointestinal tract cancers	61	3840	15.9	0.62	0.48–0.8	<0.001	0.78	0.61–1.01	0.061
Colonic polyp	19	614	31.0	1.23	0.78–1.93	0.369	1.46	0.93–2.29	0.102
Anxiety	377	12325	30.6	1.27	1.15–1.41	<0.001	1.69	1.52–1.88	<0.001
Depression	195	7018	27.8	1.15	1–1.33	0.049	1.41	1.22–1.63	<0.001
Hypothyroidism	38	1554	24.5	1.02	0.74–1.41	0.884	1.07	0.78–1.47	0.688
Parkinson’s disease	29	2284	12.7	0.48	0.34–0.7	<0.001	0.49	0.34–0.71	<0.001
Spinal cord injury	10	627	16.0	0.66	0.36–1.23	0.192	0.83	0.44–1.54	0.547

COPD: Chronic obstructive pulmonary disease.

SLE: Systemic Lupus Erythematosus.

GERD: Gastroesophageal reflux disease.

ID: Incidence density (per 1000 person-years).

^†^Multiple Cox proportional hazard regression was used to adjust for age, gender, comorbidities, and medications.

**Table 3:** Subgroup analysis of hazard ratios (95% CI) of AR for patients with and without constipation by age, gender, and comorbidities.

	Constipation		Non-constipation				
	N	No. of allergic rhinitis	N	No. of allergic rhinitis	HR	95% C.I.	p value
Age							
<20	9722	3851	10059	2246	2.09	1.98–2.2	<0.001
20–39	16677	3999	16274	1953	2.24	2.12–2.36	<0.001
40–64	16194	3130	16484	1794	2.05	1.94–2.18	<0.001
≥65	15193	2096	14969	1111	2.07	1.92–2.23	<0.001
p^†^ = 0.085							
Gender							
Female	38615	8994	38706	4793	2.13	2.05–2.2	<0.001
Male	19171	4082	19080	2311	2.02	1.92–2.13	<0.001
p^†^ = 0.154							
Hypertension							
No	48704	11684	48743	6277	2.13	2.06–2.19	<0.001
Yes	9082	1392	9043	827	1.85	1.7–2.02	<0.001
p^†^ = 0.003							
Hyperlipidemia							
No	55511	12682	55530	6856	2.11	2.04–2.17	<0.001
Yes	2275	394	2256	248	1.76	1.5–2.07	<0.001
p^†^ = 0.037							
Chronic liver disease							
No	56321	12787	56379	6927	2.10	2.04–2.17	<0.001
Yes	1465	289	1407	177	1.70	1.41–2.05	<0.001
p^†^ = 0.029							
Chronic kidney disease							
No	57351	13032	57371	7079	2.09	2.03–2.16	<0.001
Yes	435	44	415	25	1.77	1.08–2.89	0.023
p^†^ = 0.506							
Diabetes							
No	53701	12532	53766	6826	2.10	2.04–2.16	<0.001
Yes	4085	544	4020	278	2.07	1.79–2.39	<0.001
p^†^ = 0.868							
Chronic obstructive pulmonary disease							
No	56295	12746	56302	6906	2.10	2.04–2.16	<0.001
Yes	1491	330	1484	198	1.78	1.49–2.12	<0.001
p^†^ = 0.086							
Rheumatoid arthritis							
No	57638	13043	57643	7091	2.09	2.03–2.15	<0.001
Yes	148	33	143	13	2.68	1.41–5.1	0.003
p^†^ = 0.443							
Ankylosing spondylitis							
No	57749	13069	57752	7097	2.09	2.03–2.16	<0.001
Yes	37	7	34	7	0.87	0.31–2.49	0.799
p^†^ = 0.097							
Systemic lupus erythematosus							
No	57734	13062	57741	7094	2.09	2.03–2.16	<0.001
Yes	52	14	45	10	1.27	0.56–2.87	0.561
p^†^ = 0.233							
Sjogren’s syndrome							
No	57731	13058	57725	7094	2.09	2.03–2.15	<0.001
Yes	55	18	61	10	2.11	0.97–4.57	0.059
p^†^ = 0.924							
Dyspepsia							
No	57171	12912	57220	7024	2.09	2.03–2.16	<0.001
Yes	615	164	566	80	2.03	1.55–2.65	<0.001
p^†^ = 0.882							
GERD							
No	57641	13061	57659	7095	2.09	2.03–2.16	<0.001
Yes	145	15	127	9	1.53	0.67–3.5	0.313
p^†^ = 0.454							
Irritable bowel syndrome							
No	57465	12997	57505	7062	2.10	2.04–2.16	<0.001
Yes	321	79	281	42	1.70	1.17–2.47	0.006
p^†^ = 0.308							
Gastrointestinal tract cancer							
No	57297	13042	57326	7077	2.10	2.04–2.16	<0.001
Yes	489	34	460	27	1.40	0.85–2.33	0.189
p^†^ = 0.113							
Colonic polyp							
No	57720	13063	57733	7098	2.09	2.03–2.15	<0.001
Yes	66	13	53	6	1.78	0.68–4.68	0.244
p^†^ = 0.738							
Anxiety							
No	56802	12826	56855	6977	2.09	2.03–2.16	<0.001
Yes	984	250	931	127	2.08	1.68–2.57	<0.001
p^†^ = 0.974							
Depression							
No	57202	12963	57259	7022	2.10	2.04–2.16	<0.001
Yes	584	113	527	82	1.31	0.99–1.75	0.059
p^†^ = 0.001							
Hypothyroidism							
No	57664	13054	57676	7088	2.09	2.04–2.16	<0.001
Yes	122	22	110	16	1.36	0.72–2.6	0.346
p^†^ = 0.228							
Parkinson’s disease							
No	57539	13060	57536	7091	2.10	2.04–2.16	<0.001
Yes	247	16	250	13	1.14	0.55–2.36	0.733
p^†^ = 0.104							
Spinal cord injury							
No	57731	13068	57738	7102	2.09	2.03–2.15	<0.001
Yes	55	8	48	2	3.91	0.83–18.46	0.085
p^†^ = 0.467							

^†^p for difference of HR between subgroup.

## Discussion

In current study, we found a 2.3-fold higher risk of incidental AR in constipated patients than in non-constipated patients. To date and to the best of our knowledge, this is the largest and first cohort study to use a longitudinal nationwide population-based dataset to identify an increased AR risk among patients with constipation. This association may have important clinical and pathophysiological implications. Our findings highlight the considerably higher risk of AR in constipated patients. Constipation seems to be influential in development of AR. Clinicians should therefore keep a higher index of suspicion for AR in constipated patients. On the same note, constipated patients should be informed of the possible risk of AR. We also suggest that people need to maintain good bowel habits to avoid constipation that might contribute to AR.

Our findings were in line with an epidemiology study using questionnaire survey in Japan [11], including 21802 of senior high school students, aged from 15 to 18 years old, analyzing the relevant risk factors for development and remission of atopic disease. Results from this study indicated that constipated students had 1.17-fold risk for AR. However, the design of the study might have allowed some residual bias to exist and might not have controlled for possible confounding factors with propensity score methods. Similar findings were found in our study for patients under the age of 20 with a 2.09-fold AR risk. Moreover, we observed a significant higher risk of AR not only during childhood, but throughout adulthood. Consistent with our finding that there was a significant correlation between constipation and allergic diseases, Palmieri, M. et al. found a significant difference in the prevalence of atopic diseases proven by skin prick tests between the constipated children and the control group (17/52 = 33% versus 11/74 = 15%; p = 0.03) [34]. In addition, Jones et al. demonstrated that the overlap of atopy among 23471 functional gastrointestinal disorders patients, and the risk of rhinitis was 1.66-fold higher in patients with constipation than controls [8]. We also noted that hyperlipidemia, hypertension, chronic kidney disease, chronic liver disease, diabetes, COPD, RA, dyspepsia, IBS, and anxiety were associated with greater risk of AR in patients with constipation. RA was a comorbidity with a relatively higher AR risk (HR: 2.68, 95% CI, 1.41–5.1) compared with other comorbidities. Both RA and AR are characterized by the regulatory T-cells dysfunction [35]. Besides, constipation may worsen pre-existing dysbiosis in patients with RA [36, 37]. Moreover, patients with autoimmunity appeared to be predisposed to subsequent AR in our study. However, the risk for AR of other autoimmune diseases such as SLE, AS, Sjogren’s syndrome did not reach statistical significance in subgroup analysis. There might have been insufficient statistical power to detect significant differences due to the low incidence of these autoimmune diseases in our patient population.

The pathophysiology underlying the relationship between constipation and subsequent AR remains unclear. In recent times, it was shown that the gut microbiota has a pivotal role in the modulation of immunity [38–40]. Khalif et al. [17] and Feng et al. [41] reported that patients with constipation had a decreased abundance in Bifidobacteria and Lactobacillus. Khalif et al. observed that constipation was correlated with significant alterations in the fecal flora, gut permeability (leaky gut), and immune response, and that alleviation of constipation tended to reverse these changes [17]. Intestinal conditions such as constipation could affect immunity by altering gut microflora and permeability, leading to hyper-secretion of proinflammatory or inflammatory biomarkers, such as chemokines and cytokines [39, 42]. Cirali et al. found that neopterin, IL-6, and IL-12 and levels of constipated children were higher than in the non-constipation group [43], indicating that subclinical inflammation existed in patients with constipation. Mokhtare et al. also showed higher levels of TNF-α, IL-1, and IL-6in geriatric patients with chronic constipation, compared with healthy controls [42]. In experiments, TNF-α was required to produce antigen-specific IgE and to induce T-helper 2 cytokines and chemokines in allergic rhinitis [44, 45]. Moreover, gut microbiota-derived metabolites (including short-chain fatty acids such as acetate, butyrate, and propionate.) produced from high-fiber diets have been implicated as protective against allergy [46]. Trompette et al. [47] showed that mice fed low-fiber diets before nasal exposure to house dust mite extract had increased IL-4, IL-5, IL-13, and IL-17A in airway tissue, increased goblet cell hyperplasia and mucus secretion, and higher IgE levels in the serum. By contrast, mice fed high-fiber diets had a normal mucin secretion and lower cytokine levels. Moreover, fiber intake affected the intestinal microbiome composition with relative abundance of Bifidobacteriaceae and Bacteroidaceae species [47]. Low fiber intake in constipated subjects might also play a role in atopy development. Although Parthasarathy et al. have offered that mucosal microbiota analysis could discriminate 25 constipated adults from healthy controls with 94% accuracy [48]. At variance, a recent study mentioned that no disease specific separation was observed by PCoA and by calculation of diversity indices in constipated children and healthy matched controls [19]. However, both groups could be discriminated with 82% accuracy by ridge regression. Although supportive data from previous studies rarely link constipation to AR, our findings provide support to the hypothesis that constipation and AR might share a similar underlying etiological pathways related to dysbiosis. Nevertheless, the mechanism responsible for constipation-mediated dysbiosis or immune dysfunction, which precipitates AR development, requires further in-depth study.

The major advantages of this big data study were the large sample size and the relatively long time of follow-up, in which a complete history of the medical services used was available for all cases and controls. Therefore, there were minimal selection, recall, and information biases that made testing our hypothesis more feasible. Nonetheless, there were several limitations that should be noted. Firstly, the NHIRD does not disclose information regarding the patients’ diet, socioeconomic status, family history, personal lifestyle, such as smoking, alcohol drinking, and dietary preference, and environmental exposures, which may be associated risk factors for development of AR. Although we adjusted for a variety of comorbidities and medications, and matched propensity scores to reduce the confounders, unmeasured factors might have biased our results. Secondly, the diagnoses of constipation, AR, and comorbidities were entirely dependent on the ICD-9 codes in the administrative dataset. Therefore, the accuracy of diagnoses could not be verified by personal review of medical records and this may have resulted in misclassification. It is worth noting that these misclassifications were more likely to be random, and associations are often underestimated rather than overestimated. However, Taiwan’s NHI administration has set up an ad hoc committee to monitor the accuracy of claimed data to prevent violations. Furthermore, we only selected subjects that were repeatedly coded to increase the validity and accuracy of the diagnoses. Thirdly, relevant clinical information, such as laboratory data including cytokines and gene-expression changes, imaging findings, and fecal microbiota assessments were unavailable in the database and therefore could not be included in the analyses.

There is mounting evidence showing that fecal microbiota transplantation (FMT) may be an effective therapeutic approach for intractable constipation [20, 21, 41]. This implies that intestinal dysbiosis is causally related to the pathogenesis or a consequence of constipation. Modulation of the intestinal flora to restore a diverse and balanced microbiome may treat or prevent microbiota-related disease. Furthermore, while constipation can be partially controlled with laxatives, but the disrupted gut microflora may not be completely changed. Therefore, as AR appears to be mediated, at least in part, through the microbiota-gut-airway axis, other therapeutic possibilities for constipation should be considered, such as use of probiotics, prebiotics, synbiotics, and FMT to restore the intestinal flora. Our research provides an observational evidence of an association between constipation and AR. Further research should be conducted to determine how constipation changes the composition of the gut microbiota and the extent to which this affects AR. We also speculate that relief of constipation in patients with AR might be helpful when used in combination with other approaches. State-of-the-art metagenomic and metabolomic analyses of the gut microbiota in constipated patients are needed to better understand their interactions with the immune system.

## Conclusion

The risk of AR in constipated patients is twice the risk in non-constipated patients. It seems that physicians should keep a higher index of suspicion for AR in people with constipation. The patency issue of gut could not be ignored in patients with AR. Further comprehensive basic and clinical research is needed to elucidate the mechanisms underlying these associations.
