# Supplementary material for: Constipation might be associated with risk of allergic rhinitis: A nationwide population-based cohort study
Source: PLoS One. 2020 Oct 2;15(10):e0239723. doi: 10.1371/journal.pone.0239723 (PMC7531808; doi:10.1371/journal.pone.0239723)
Supplement: Attachment — Submitted filename: O-2020-002317 R1 Response to Reviewers20200629.docx [file pone.0239723.s001.docx]

Tomohiko Ai, M.D., Ph.D.

Academic Editor

**Journal**: PLOS ONE

Dear Dr. Tomohiko Ai

On behalf of my coauthors, I thank the Editor/Reviewers for the comments and am grateful for the opportunity to revise our manuscript (ID: PONE-D-20-09268) titled, “Constipation is associated with risk of allergic rhinitis: A nationwide population-based cohort study”, for consideration of publication as an Original Research in PLOS ONE. Your comments were highly insightful and enabled us to improve the quality of our document. In the following pages are our responses to each comment.

Revisions in the text are shown [yellow highlights]. We have addressed the comments point-by-point, and the corresponding changes have been made in track change in the manuscript, which you will find uploaded alongside this document. We hope that our revisions to the document combined with our accompanying responses will be sufficient to render our document suitable for publication in PLOS ONE.

We look forward to hearing from you.

Kind regards,

James Cheng-Chung Wei, MD, PhD

Institute of Medicine, Chung Shan Medical University

No. 110, Sec 1, Jianguo N. Road, Taichung, 40201, Taiwan

jccwei@gmail.com

**Responses to the Editors/Reviewers’ Comments:**

**Manuscript ID:** **PONE-D-20-09268**

**Manuscript title:** Constipation is associated with risk of allergic rhinitis: A nationwide population-based cohort study

**Journal: PLOS ONE**

Editor/Reviewer(s)' Comments to Author:

**1.** There are many factors that are associated with constipation: various GI diseases such as cancers and polyps; imbalance in immune and nervous system function, bile acid metabolism and mucus secretion, and the gastrointestinal microbiota and fermentation (e.g., Nat Rev Dis Primers. 2017 Dec 14;3:17095).

The authors claimed that the high prevalence of constipation is a risk factor for allergic rhinitis. But, any supporting data are not shown to link these two conditions. Just statistical findings cannot prove your hypothesis (maybe coincidental).

**Response:**

Thank you for your comments and suggestions.

We analyzed a variety of comorbid conditions and medications in detail that were associated with constipation based on the reference (Nat Rev Dis Primers. 2017 Dec 14;3:17095). We considered the most chronic gastrointestinal conditions, such as dyspepsia, GERD, IBS, colonic polyps, GI tract cancers. Psychiatric/Mood disorders such as anxiety and depression; autoimmune diseases including SLE, RA, AS, Sjogren’s syndrome; neurological diseases such as Parkinson’s disease, multiple sclerosis, spinal cord injury and hypothyroidism were also enrolled for analysis. In addition, we further considered various medications associated with constipation including non-steroidal anti-inflammatory drugs, calcium channel blockers, diuretics, opioids, antidepressants, serotonin antagonists, anticonvulsants, antispasmodic, iron supplement, calcium supplement. Furthermore, we attempted to use propensity score matching to achieve more complete control of the potential confounders.

We added this paragraph for explanation as below in method section:

The baseline characteristics were age, gender, related comorbidities including hyperlipidemia (ICD-9-CM=272.0-272.4), hypertension (ICD-9-CM=401-405), chronic kidney disease (ICD-9-CM=585), chronic liver disease (ICD-9-CM=571), diabetes (ICD-9-CM=250), chronic obstructive pulmonary disease (COPD) (ICD-9-CM =491, 492, 496), autoimmune diseases such as systemic lupus erythematosus (SLE) (ICD-9-CM=710.0), rheumatoid arthritis (RA) (ICD-9-CM=714.0), Sjogren’s syndrome (ICD-9-CM=710.2), ankylosing spondylitis (AS) (ICD-9-CM=720.0), and the diseases predisposing constipation [31], including dyspepsia (ICD-9-CM=536.8), gastroesophageal reflux disease (GERD) (ICD-9-CM=530.11, 530.8x), irritable bowel syndrome (IBS) (ICD-9-CM=564.1), gastrointestinal tract cancers (ICD-9-CM=150-159), colonic polyp (ICD-9-CM=V12.72, 211.3 and 211.4), anxiety (ICD-9-CM=300.0), depression (ICD-9-CM=296.2, 296.3, 300.4 and 311), hypothyroidism (ICD-9-CM=243, 244), Parkinson’s disease (ICD-9-CM=322), multiple sclerosis (ICD-9-CM=340), spinal cord injury (ICD-9-CM=806, 952). The comorbidities were defined as occurring within one year prior to the index date and with at least three outpatient visits or one hospitalization. In addition, medications containing corticosteroids, antihistamines, non-steroidal anti-inflammatory drugs, calcium channel blockers, diuretics, opioids, antidepressants, serotonin antagonists, anticonvulsants, antispasmodic, iron supplement, calcium supplement during the study period were included and defined as usage for ≥30 days.

Propensity-score matching was performed to match the two groups based on age, gender, hypertension, hyperlipidemia, chronic liver disease, diabetes, chronic kidney disease, COPD, SLE, RA, Sjogren’s syndrome, AS, dyspepsia, GERD, IBS, gastrointestinal tract cancers, colonic polyp, anxiety, depression, hypothyroidism, Parkinson’s disease, multiple sclerosis, spinal cord injury, and various medications.

We have added new revised tables 1-3 (Attached at the end of the response letter), revised figure 1 and revised figure 2 in the revised manuscript:

Fig 1. Flowchart of study.

Fig 2. Kaplan–Meier curves of the cumulative probability of AR in the study groups.

We revised in results section as below and also revised in the abstract:

We identified 57786 patients with constipation and 57786 matched controls between 1999 and 2013 from the LHID, a subset of Taiwanese NHIRD………………………..

…………………………………………………………………………………………Table 2 displays the incidence density and risk factors for AR. The incidence of AR was 32.2 per 1,000 person-years in constipation patients, which was higher than the rate of 14.8 per 1,000 person-years found in non-constipation patients. After adjustment, patients with constipation had a significantly higher risk of AR than those without constipation (aHR, 2.30; 95% CI, 2.23-2.37; P < 0.001), and older age groups were associated with a lower risk of developing AR when compared with the <20 years age group. Compared with women, men had a higher risk of AR (aHR, 1.07; 95% CI, 1.04-1.10; P < 0.001). In terms of comorbidities, patients with hypertension (aHR, 1.61; 95% CI, 1.52-1.71; P < 0.001), hyperlipidemia (aHR, 1.23; 95% CI, 1.13-1.34; P < 0.001), chronic liver disease (aHR, 1.44; 95% CI, 1.31-1.58; P < 0.001), COPD (aHR, 2.02; 95% CI, 1.84-2.21; P < 0.001), RA (aHR, 1.58; 95% CI, 1.18-2.12; P =0.002), AS (aHR, 1.81; 95% CI, 1.07-3.06; P=0.027), SLE (aHR, 1.88; 95% CI, 1.25-2.83; P=0.002), Sjogren’s syndrome (aHR, 2.45; 95% CI, 1.68-3.58; P < 0.001), dyspepsia (aHR, 1.34; 95% CI, 1.18-1.52; P < 0.001), IBS (aHR, 1.45; 95% CI, 1.21-1.73; P < 0.001), anxiety (aHR, 1.69; 95% CI, 1.52-1.88; P < 0.001) and depression (aHR, 1.41; 95% CI, 1.22-1.63; P < 0.001) were at higher risk of AR. In Table 3, stratified analyses were performed to assess the relationship between constipation and AR based on demographic characteristics. In patients aged 20-39 years, compared with the non-constipation cohort, there was a 2.24-fold higher risk of AR in the constipation cohort (aHR; 95% CI, 2.12-2.36; P < 0.001). Patients aged <20, 40-64, and ≥65 years had a 2.09, 2.05, and 2.07-fold risk of AR in the constipation cohort (aHR; 95% CI, 1.98-2.20, 1.94-2.18 and 1.92-2.23; P < 0.001), respectively. Among women, compared with patients without constipation, there was a 2.13-fold higher risk of AR in patients with constipation (aHR; 95% CI, 2.05-2.20; P < 0.001). Among men, there was 2.02-fold higher risk of AR in patients with constipation (aHR; 95% CI, 1.92-2.13; P < 0.001). Furthermore, patients with constipation had a significantly higher likelihood of AR, regardless of sex, and with or without comorbidities including hyperlipidemia, hypertension, chronic kidney disease, chronic liver disease, diabetes, COPD, RA, dyspepsia, IBS, and anxiety. …………………………………………………………………………………

From the point of view of the levels of evidence in evidence-based medicine, to date, there have been no meta-analyses, randomized control trials, or cohort studies related to constipation and AR risk. There are only a few small-scale observational studies to support these associations in the literature. Considering that supportive data from previous studies rarely link constipation to AR, our study aimed to investigate the association between constipation and AR by a large nationwide population-based cohort design. We tried to overcome the limitations of previous studies by using a large sample size, adjusting for various comorbidities and a variety of medications, matching propensity scores to reduce potential confounding factors, which made it feasible to test our hypothesis. Such research is of importance to provide evidence for further basic and clinical research. We also included a shred of studies have suggested that atopy might be linked with constipation, and cited more evidences to support and reinforce the hypothesis of current research.

We revised the paragraph in introduction and in discussion section and added the sentences as below:

In 2^nd^ paragraph of introduction section:

A shred of studies have suggested that atopic disease might be linked to constipation [6-8]. Studies have reported an indirect indication of a concurrence of constipation and atopy by demonstrating a high prevalence of coexistent allergic manifestations in constipated children investigated for cow milk allergy [9-10]. An epidemiologic survey by Tokunaga et al. showed that constipation was a relevant factor for AR development (adjusted odds ratio of 1.17) among 21802 high school students [11]. Jones et al. stated that the overlap of atopy in functional gastrointestinal disorders patients, and the risk of rhinitis in 342 constipated patients was 1.66 times higher than controls [8]. …………………………………………………………………………….

………………………………………………………………………………………….

In 3^rd^ paragraph of discussion section:

………………………………………………………………………………………….…………………………………………………………………………………………. Although Parthasarathy et al. have offered that mucosal microbiota analysis could discriminate 25 constipated adults from healthy controls with 94% accuracy [47]. At variance, a recent study mentioned that no disease specific separation was observed by PCoA and by calculation of diversity indices in constipated children and healthy matched controls [19]. However, both groups could be discriminated with 82% accuracy by ridge regression. Although supportive data from previous studies rarely link constipation to AR, our findings provide support to the hypothesis that constipation and AR might share a similar underlying etiological pathways related to dysbosis. Nevertheless, the mechanism responsible for constipation-mediated dysbiosis or immune dysfunction, which precipitates AR development, requires further in-depth study.

I greatly appreciate your time and your kind comments again; your comments reinforced our manuscript and we did our best to respond to your comments carefully.

**2.** In Discussion, the authors described a lot about the results and speculations by other researchers’ studies. However, there are no discussion how these can support your data (i.e., the authors describe others’ speculations to explain your speculations). Please discuss about the presented data.

**Response:**

Thank you for your suggestions.

We had revised and discussed presented data in more detail. We added this paragraph for explanation as below in 2^nd^ paragraph of discussion section:

………………………………………………………………………………………….…………………………………………………………………………………………

Consistent with our finding that there was a significant correlation between constipation and allergic diseases, Palmieri, M. et al. found a significant difference in the prevalence of atopic diseases proven by skin prick tests between the constipated children and the control group (17/52 = 33% versus 11/74=15%; p = 0.03) [33]. In addition, Jones et al. demonstrated that the overlap of atopy among 23471 functional gastrointestinal disorders patients, and the risk of rhinitis was 1.66-fold higher in patients with constipation than controls [8]. We also noted that hyperlipidemia, hypertension, chronic kidney disease, chronic liver disease, diabetes, COPD, RA, dyspepsia, IBS, and anxiety were associated with greater risk of AR in patients with constipation. RA was a comorbidity with a relatively higher AR risk (HR: 2.68, 95% CI, 1.41-5.1) compared with other comorbidities. Both RA and AR are characterized by the regulatory T-cells dysfunction [34]. Besides, constipation may worsen pre-existing dysbiosis in patients with RA [35, 36]. Moreover, patients with autoimmunity appeared to be predisposed to subsequent AR in our study. However, the risk for AR of other autoimmune diseases such as SLE, AS, Sjogren’s syndrome did not reach statistical significance in subgroup analysis. There might have been insufficient statistical power to detect significant differences due to the low incidence of these autoimmune diseases in our patient population.

It has been mentioned in other studies that dysbiotic microbiome could affect both constipation and AR, therefore we speculated the possibility that this could be a mechanism behind the association. We have included in the discussion that it is a limitation that we do not have fecal microbiota assessment, but more research can be done to assess this possibility.

**3.** Also, the background of study participants varies. For example, the authors included autoimmune diseases. Please describe more details (e.g., types of diseases), and explain or speculate how each autoimmune disease can be associated with AR and/or constipation (i.e., pathophysiological mechanisms).

**Response:**

Thank you for your comments. We clarify the types of autoimmune diseases including systemic lupus erythematosus (SLE), rheumatoid arthritis (RA), Sjogren’s syndrome, ankylosing spondylitis (AS) and found that patients with autoimmunity appeared to be predisposed to subsequent AR in our study (as shown in table 2). RA was a comorbidity with a relatively higher AR risk (HR: 2.68, 95% CI, 1.41-5.1) compared with other comorbidities. We also explain the possible pathophysiological mechanisms as below in discussion:

……………………………………………………………………………………………………………………………………………………………………………………RA was a comorbidity with a relatively higher AR risk (HR: 2.68, 95% CI, 1.41-5.1) compared with other comorbidities. Both RA and AR are characterized by the regulatory T-cells dysfunction [34]. Besides, constipation may worsen pre-existing dysbiosis in patients with RA [35, 36]. Moreover, patients with autoimmunity appeared to be predisposed to subsequent AR in our study. However, the risk for AR of other autoimmune diseases such as SLE, AS, Sjogren’s syndrome did not reach statistical significance in subgroup analysis. There might have been insufficient statistical power to detect significant differences due to the low incidence of these autoimmune diseases in our patient population.

We further cited reference as below:

34.	Cooles FA, Isaacs JD, Anderson AE. Treg cells in rheumatoid arthritis: an update. Curr Rheumatol Rep. 2013;15(9):352.

35.	Häger J, Bang H, Hagen M, Frech M, Träger P, Sokolova MV, et al. The Role of Dietary Fiber in Rheumatoid Arthritis Patients: A Feasibility Study. Nutrients. 2019;11(10).

36.	Horta-Baas G, Romero-Figueroa MDS, Montiel-Jarquín AJ, Pizano-Zárate ML, García-Mena J, Ramírez-Durán N. Intestinal Dysbiosis and Rheumatoid Arthritis: A Link between Gut Microbiota and the Pathogenesis of Rheumatoid Arthritis. J Immunol Res. 2017;2017:4835189.

**4.** I would request the authors to present more detail data showing direct, at least suggestive, evidences for your hypothesis (i.e., underlying mechanisms linking constipation and AR).

**Response:**

We further analyzed a variety of associated comorbidities and various medications (based on Nat Rev Dis Primers. 2017 Dec 14;3:17095) and matched propensity scores to reduce the bias of possible residual confounders. We found a significantly higher risk of AR (aHR, **2.30**; 95% CI, 2.23-2.37) was observed in constipated cohort than non-constipated cohort and constipated patients had a significantly higher likelihood of AR, regardless of age, sex, and with or without comorbidities including hyperlipidemia, hypertension, chronic kidney disease, chronic liver disease, diabetes, COPD, RA, dyspepsia, IBS, and anxiety. We have presented more detail data in the revised manuscript. The association between constipation and AR have been suggested in a few studies. [8, 11, 33] We further cited more-recent studies to offer the evidences regarding constipation and dysbiosis in adults and children. In addition, some up-to-date research of dysbiotic fecal microbiota in AR patients has been added to support the hypothesis that constipation and AR might share potential etiological pathways related to dysbiosis. We revised the paragraph of discussion regarding possible pathophysiological mechanisms as below. However, the observational design of current research precluded to elucidate the mechanisms underlying these associations, this is an inherent limitation of population-based datasets such as the NHIRD, future studies are warranted to validate our findings. (such as state-of-the-art metagenomic and metabolomic analyses of the gut microbiota in constipated patients are warranted to elucidate the possible pathogenetic mechanisms underlying these associations.)

In 2^nd^ paragraph of discussion section:

…………………………………………………………………………………………………………………………………………………………………………………….. Consistent with our finding that there was a significant correlation between constipation and allergic diseases, Palmieri, M. et al. found a significant difference in the prevalence of atopic diseases proven by skin prick tests between the constipated children and the control group (17/52 = 33% versus 11/74=15%; p = 0.03) [33]. In addition, Jones et al. demonstrated that the overlap of atopy among 23471 functional gastrointestinal disorders patients, and the risk of rhinitis was 1.66-fold higher in patients with constipation than controls [8]. We also noted that hyperlipidemia, hypertension, chronic kidney disease, chronic liver disease, diabetes, COPD, RA, dyspepsia, IBS, and anxiety were associated with greater risk of AR in patients with constipation……………………………………………………………………………..

In 3^rd^ paragraph of discussion section:

The pathophysiology underlying the relationship between constipation and subsequent AR remain ………………………………………………………................

.………………………………………………………………………………………….…………………………………………………………………………………………..Although Parthasarathy et al. have offered that mucosal microbiota analysis could discriminate 25 constipated adults from healthy contols with 94% accuracy [47]. At variance, a recent study mentioned that no disease specific separation was observed by PCoA and by calculation of diversity indices in constipated children and healthy matched controls [19]. However, both groups could be discriminated with 82% accuracy by ridge regression. Although supportive data from previous studies rarely link constipation to AR, our findings provide support to the hypothesis that constipation and AR might share a similar underlying etiological pathways related to dysbiosis. Nevertheless, the mechanism responsible for constipation-mediated dysbiosis or immune dysfunction, which precipitates AR development, requires further in-depth study.

8.	Jones MP, Walker MM, Ford AC, Talley NJ. The overlap of atopy and functional gastrointestinal disorders among 23,471 patients in primary care. Aliment Pharmacol Ther. 2014;40: 382-391.

11.	Tokunaga T, Ninomiya T, Osawa Y, Imoto Y, Ito Y, Takabayashi T, et al. Factors associated with the development and remission of allergic diseases in an epidemiological survey of high school students in Japan. Am J Rhinol Allergy. 2015;29(2): 94-99.

33.	Palmieri M, Ardia E, Caro MA, Palmieri S. Constipation and atopic diseases in children. Italian Journal of Allergy and Clinical Immunology. 2009;19:39-43.

Besides, we further cited reference as below:

19.	de Meij TG, de Groot EF, Eck A, Budding AE, Frank Kneepkens CM, Benninga MA, et al. Characterization of microbiota in children with chronic functional constipation. PLoS One. 2016;11(10): e0164731.

24.	Liu X, Tao J, Li J, Cao X, Li Y, Gao X, et al. Dysbiosis of Fecal Microbiota in Allergic Rhinitis Patients. Am J Rhinol Allergy. 2020:1945892420920477.

27.	Kim WG, Kang GD, Kim HI, Han MJ, Kim DH. Bifidobacterium longum IM55 and Lactobacillus plantarum IM76 alleviate allergic rhinitis in mice by restoring Th2/Treg imbalance and gut microbiota disturbance. Benef Microbes. 2019;10(1):55-67.

47.	Parthasarathy G, Chen J, Chen X, Chia N, O'Connor HM, Wolf PG, et al. Relationship Between Microbiota of the Colonic Mucosa vs Feces and Symptoms, Colonic Transit, and Methane Production in Female Patients With Chronic Constipation. Gastroenterology. 2016;150(2):367-79.

We thank again from you for your best comments. Dear editor, your comment helps us to promote quality of this manuscript and we appreciate your kind suggestions.

We have add new Tables 1-3 as below:

**Table 1. Demographic characteristics of constipation group and non-constipation group.**

|  | Before propensity score matched | | | | |  |  | After propensity score matched | | | | |  |
| --- | --- | --- | --- | --- | --- | --- | --- | --- | --- | --- | --- | --- | --- |
|  | Constipation (N =75500) | |  | Non-constipation (N =75500) | |  |  | Constipation (N =57786) | |  | Non-constipation (N =57786) | |  |
|  | n | % |  | n | % | ASD |  | n | % |  | n | % | ASD |
| Age |  | |  |  | | <0.001 |  |  |  |  |  |  | 0.026 |
| <20 | 11001 | 14.6 |  | 11001 | 14.6 |  |  | 9722 | 16.8 |  | 10059 | 17.4 |  |
| 20-39 | 20395 | 27.0 |  | 20395 | 27.0 |  |  | 16677 | 28.9 |  | 16274 | 28.2 |  |
| 40-64 | 22337 | 29.6 |  | 22337 | 29.6 |  |  | 16194 | 28.0 |  | 16484 | 28.5 |  |
| ≥65 | 21767 | 28.8 |  | 21767 | 28.8 |  |  | 15193 | 26.3 |  | 14969 | 25.9 |  |
| Mean ± SD | 46.4 ± 23.6 | |  | 46.4 ± 23.6 | | <0.001 |  | 44.4 ± 24 | |  | 44 ± 23.8 | | 0.018 |
| Gender |  |  |  |  |  | <0.001 |  |  |  |  |  |  | 0.003 |
| Female | 49243 | 65.2 |  | 49243 | 65.2 |  |  | 38615 | 66.8 |  | 38706 | 67.0 |  |
| Male | 26257 | 34.8 |  | 26257 | 34.8 |  |  | 19171 | 33.2 |  | 19080 | 33.0 |  |
| Hypertension | 15253 | 20.2 |  | 10458 | 13.9 | 0.170 |  | 9082 | 15.7 |  | 9043 | 15.6 | 0.002 |
| Hyperlipidemia | 3938 | 5.2 |  | 2615 | 3.5 | 0.086 |  | 2275 | 3.9 |  | 2256 | 3.9 | 0.002 |
| Chronic liver disease | 3058 | 4.1 |  | 1497 | 2.0 | 0.121 |  | 1465 | 2.5 |  | 1407 | 2.4 | 0.006 |
| Chronic kidney disease | 883 | 1.2 |  | 493 | 0.7 | 0.054 |  | 435 | 0.8 |  | 415 | 0.7 | 0.004 |
| Diabetes | 7808 | 10.3 |  | 4387 | 5.8 | 0.167 |  | 4085 | 7.1 |  | 4020 | 7.0 | 0.004 |
| COPD | 3128 | 4.1 |  | 1573 | 2.1 | 0.119 |  | 1491 | 2.6 |  | 1484 | 2.6 | 0.001 |
| Rheumatoid arthritis | 281 | 0.4 |  | 165 | 0.2 | 0.028 |  | 148 | 0.3 |  | 143 | 0.2 | 0.002 |
| Ankylosing spondylitis | 92 | 0.1 |  | 34 | 0.0 | 0.027 |  | 37 | 0.1 |  | 34 | 0.1 | 0.002 |
| SLE | 75 | 0.1 |  | 60 | 0.1 | 0.007 |  | 52 | 0.1 |  | 45 | 0.1 | 0.004 |
| Sjogren's syndrome | 124 | 0.2 |  | 65 | 0.1 | 0.022 |  | 55 | 0.1 |  | 61 | 0.1 | 0.003 |
| Dyspepsia | 1867 | 2.5 |  | 567 | 0.8 | 0.137 |  | 615 | 1.1 |  | 566 | 1.0 | 0.008 |
| GERD | 528 | 0.7 |  | 127 | 0.2 | 0.081 |  | 145 | 0.3 |  | 127 | 0.2 | 0.006 |
| Irritable bowel syndrome | 955 | 1.3 |  | 281 | 0.4 | 0.099 |  | 321 | 0.6 |  | 281 | 0.5 | 0.010 |
| Gastrointestinal tract cancers | 905 | 1.2 |  | 502 | 0.7 | 0.056 |  | 489 | 0.8 |  | 460 | 0.8 | 0.006 |
| Colonic polyp | 170 | 0.2 |  | 53 | 0.1 | 0.040 |  | 66 | 0.1 |  | 53 | 0.1 | 0.007 |
| Anxiety | 2566 | 3.4 |  | 956 | 1.3 | 0.142 |  | 984 | 1.7 |  | 931 | 1.6 | 0.007 |
| Depression | 1988 | 2.6 |  | 534 | 0.7 | 0.151 |  | 584 | 1.0 |  | 527 | 0.9 | 0.010 |
| Hypothyroidism | 213 | 0.3 |  | 117 | 0.2 | 0.027 |  | 122 | 0.2 |  | 110 | 0.2 | 0.005 |
| Parkinson’s disease | 879 | 1.2 |  | 250 | 0.3 | 0.097 |  | 247 | 0.4 |  | 250 | 0.4 | 0.001 |
| Multiple sclerosis | 10 | 0.0 |  | 2 | 0.0 | 0.012 |  | 1 | 0.0 |  | 2 | 0.0 | 0.003 |
| Spinal cord injury | 267 | 0.4 |  | 48 | 0.1 | 0.064 |  | 55 | 0.1 |  | 48 | 0.1 | 0.004 |
| Corticosteroids | 20240 | 26.8 |  | 12612 | 16.7 | 0.247 |  | 11729 | 20.3 |  | 11673 | 20.2 | 0.002 |
| Antihistamines | 51295 | 67.9 |  | 38014 | 50.3 | 0.364 |  | 35663 | 61.7 |  | 35677 | 61.7 | <0.001 |
| NSAIDs | 55459 | 73.5 |  | 43706 | 57.9 | 0.332 |  | 39317 | 68.0 |  | 39457 | 68.3 | 0.005 |
| Calcium channel blockers | 21533 | 28.5 |  | 16949 | 22.4 | 0.140 |  | 13656 | 23.6 |  | 13556 | 23.5 | 0.004 |
| Diuretics | 18886 | 25.0 |  | 12421 | 16.5 | 0.212 |  | 10842 | 18.8 |  | 10868 | 18.8 | 0.001 |
| Opioids | 9228 | 12.2 |  | 4973 | 6.6 | 0.194 |  | 4657 | 8.1 |  | 4583 | 7.9 | 0.005 |
| Antidepressants | 14216 | 18.8 |  | 5566 | 7.4 | 0.345 |  | 5698 | 9.9 |  | 5455 | 9.4 | 0.014 |
| Serotonin (5HT3) antagonists | 772 | 1.0 |  | 472 | 0.6 | 0.044 |  | 428 | 0.7 |  | 444 | 0.8 | 0.003 |
| Anticonvulsants | 11968 | 15.9 |  | 4833 | 6.4 | 0.304 |  | 4910 | 8.5 |  | 4681 | 8.1 | 0.014 |
| Antispasmodic | 19669 | 26.1 |  | 11406 | 15.1 | 0.273 |  | 10934 | 18.9 |  | 10894 | 18.9 | 0.002 |
| Iron supplement | 4229 | 5.6 |  | 2365 | 3.1 | 0.121 |  | 2212 | 3.8 |  | 2176 | 3.8 | 0.003 |
| Calcium supplement | 7654 | 10.1 |  | 4677 | 6.2 | 0.144 |  | 4186 | 7.2 |  | 4153 | 7.2 | 0.002 |

ASD: Absolute standardized differences.

**Table 2. Multiple Cox proportional hazard regression for the estimation of adjusted hazard ratios for AR.**

|  | No. of allergic rhinitis | Observed Person-Years | ID | Crude HR | 95% C.I. | p value | Adjusted HR^†^ | 95% C.I. | p value |
| --- | --- | --- | --- | --- | --- | --- | --- | --- | --- |
| Group |  |  |  |  |  |  |  |  |  |
| Non-constipation | 7104 | 480068 | 14.8 | 1 |  |  | 1 |  |  |
| Constipation | 13076 | 405708 | 32.2 | 2.09 | 2.03-2.15 | <0.001 | 2.30 | 2.23-2.37 | <0.001 |
| Age |  |  |  |  |  |  |  |  |  |
| <20 | 6097 | 163913 | 37.2 | 1 |  |  | 1 |  |  |
| 20-39 | 5952 | 270959 | 22.0 | 0.57 | 0.55-0.59 | <0.001 | 0.58 | 0.56-0.6 | <0.001 |
| 40-64 | 4924 | 262465 | 18.8 | 0.48 | 0.47-0.5 | <0.001 | 0.62 | 0.6-0.65 | <0.001 |
| ≥65 | 3207 | 188439 | 17.0 | 0.41 | 0.4-0.43 | <0.001 | 0.65 | 0.62-0.69 | <0.001 |
| Gender |  |  |  |  |  |  |  |  |  |
| Female | 13787 | 617078 | 22.3 | 1 |  |  | 1 |  |  |
| Male | 6393 | 268699 | 23.8 | 1.04 | 1.01-1.07 | 0.022 | 1.07 | 1.04-1.1 | <0.001 |
| Hypertension | 2219 | 113499 | 19.6 | 0.78 | 0.75-0.82 | <0.001 | 1.61 | 1.52-1.71 | <0.001 |
| Hyperlipidemia | 642 | 28879 | 22.2 | 0.91 | 0.84-0.98 | 0.014 | 1.23 | 1.13-1.34 | <0.001 |
| Chronic liver disease | 466 | 17869 | 26.1 | 1.10 | 1-1.2 | 0.048 | 1.44 | 1.31-1.58 | <0.001 |
| Chronic kidney disease | 69 | 3696 | 18.7 | 0.71 | 0.56-0.9 | 0.004 | 0.95 | 0.75-1.21 | 0.678 |
| Diabetes | 822 | 47744 | 17.2 | 0.69 | 0.64-0.73 | <0.001 | 0.86 | 0.8-0.93 | <0.001 |
| COPD | 528 | 13425 | 39.3 | 1.55 | 1.42-1.69 | <0.001 | 2.02 | 1.84-2.21 | <0.001 |
| Rheumatoid arthritis | 46 | 1887 | 24.4 | 1.04 | 0.78-1.39 | 0.783 | 1.58 | 1.18-2.12 | 0.002 |
| Ankylosing spondylitis | 14 | 475 | 29.5 | 1.24 | 0.74-2.1 | 0.412 | 1.81 | 1.07-3.06 | 0.027 |
| SLE | 24 | 649 | 37.0 | 1.55 | 1.04-2.32 | 0.031 | 1.88 | 1.25-2.83 | 0.002 |
| Sjogren’s syndrome | 28 | 653 | 42.9 | 1.71 | 1.18-2.48 | 0.005 | 2.45 | 1.68-3.58 | <0.001 |
| Dyspepsia | 244 | 7542 | 32.4 | 1.37 | 1.21-1.55 | <0.001 | 1.34 | 1.18-1.52 | <0.001 |
| GERD | 24 | 1283 | 18.7 | 0.68 | 0.45-1.01 | 0.056 | 0.59 | 0.4-0.88 | 0.010 |
| Irritable bowel syndrome | 121 | 3789 | 31.9 | 1.35 | 1.13-1.61 | 0.001 | 1.45 | 1.21-1.73 | <0.001 |
| Gastrointestinal tract cancers | 61 | 3840 | 15.9 | 0.62 | 0.48-0.8 | <0.001 | 0.78 | 0.61-1.01 | 0.061 |
| Colonic polyp | 19 | 614 | 31.0 | 1.23 | 0.78-1.93 | 0.369 | 1.46 | 0.93-2.29 | 0.102 |
| Anxiety | 377 | 12325 | 30.6 | 1.27 | 1.15-1.41 | <0.001 | 1.69 | 1.52-1.88 | <0.001 |
| Depression | 195 | 7018 | 27.8 | 1.15 | 1-1.33 | 0.049 | 1.41 | 1.22-1.63 | <0.001 |
| Hypothyroidism | 38 | 1554 | 24.5 | 1.02 | 0.74-1.41 | 0.884 | 1.07 | 0.78-1.47 | 0.688 |
| Parkinson’s disease | 29 | 2284 | 12.7 | 0.48 | 0.34-0.7 | <0.001 | 0.49 | 0.34-0.71 | <0.001 |
| Spinal cord injury | 10 | 627 | 16.0 | 0.66 | 0.36-1.23 | 0.192 | 0.83 | 0.44-1.54 | 0.547 |

ID: Incidence density (per 1000 person-years).

†Adjusted for age, gender, comorbidities, and medications.

**Table 3. Subgroup analysis of adjusted hazard ratios (95% CI) stratified by age, gender, and comorbidities.**

|  | Constipation | |  | Non-constipation | |  |  |  |
| --- | --- | --- | --- | --- | --- | --- | --- | --- |
|  | N | No. of allergic rhinitis |  | N | No. of allergic rhinitis | HR | 95% C.I. | p value |
| Age |  |  |  |  |  |  |  |  |
| <20 | 9722 | 3851 |  | 10059 | 2246 | 2.09 | 1.98-2.2 | <0.001 |
| 20-39 | 16677 | 3999 |  | 16274 | 1953 | 2.24 | 2.12-2.36 | <0.001 |
| 40-64 | 16194 | 3130 |  | 16484 | 1794 | 2.05 | 1.94-2.18 | <0.001 |
| ≥65 | 15193 | 2096 |  | 14969 | 1111 | 2.07 | 1.92-2.23 | <0.001 |
| p for interaction= 0.085 | | | | | | | | |
| Gender |  |  |  |  |  |  |  |  |
| Female | 38615 | 8994 |  | 38706 | 4793 | 2.13 | 2.05-2.2 | <0.001 |
| Male | 19171 | 4082 |  | 19080 | 2311 | 2.02 | 1.92-2.13 | <0.001 |
| p for interaction= 0.154 | | | | | | | | |
| Hypertension |  |  |  |  |  |  |  |  |
| No | 48704 | 11684 |  | 48743 | 6277 | 2.13 | 2.06-2.19 | <0.001 |
| Yes | 9082 | 1392 |  | 9043 | 827 | 1.85 | 1.7-2.02 | <0.001 |
| p for interaction= 0.003 | | | | | | | | |
| Hyperlipidemia |  |  |  |  |  |  |  |  |
| No | 55511 | 12682 |  | 55530 | 6856 | 2.11 | 2.04-2.17 | <0.001 |
| Yes | 2275 | 394 |  | 2256 | 248 | 1.76 | 1.5-2.07 | <0.001 |
| p for interaction= 0.037 | | | | | | | | |
| Chronic liver disease | |  |  |  |  |  |  |  |
| No | 56321 | 12787 |  | 56379 | 6927 | 2.10 | 2.04-2.17 | <0.001 |
| Yes | 1465 | 289 |  | 1407 | 177 | 1.70 | 1.41-2.05 | <0.001 |
| p for interaction= 0.029 | | | | | | | | |
| Chronic kidney disease | |  |  |  |  |  |  |  |
| No | 57351 | 13032 |  | 57371 | 7079 | 2.09 | 2.03-2.16 | <0.001 |
| Yes | 435 | 44 |  | 415 | 25 | 1.77 | 1.08-2.89 | 0.023 |
| p for interaction= 0.506 | | | | | | | | |
| Diabetes |  |  |  |  |  |  |  |  |
| No | 53701 | 12532 |  | 53766 | 6826 | 2.10 | 2.04-2.16 | <0.001 |
| Yes | 4085 | 544 |  | 4020 | 278 | 2.07 | 1.79-2.39 | <0.001 |
| p for interaction= 0.868 | | | | | | | | |
| Chronic obstructive pulmonary disease | | |  |  |  |  |  |  |
| No | 56295 | 12746 |  | 56302 | 6906 | 2.10 | 2.04-2.16 | <0.001 |
| Yes | 1491 | 330 |  | 1484 | 198 | 1.78 | 1.49-2.12 | <0.001 |
| p for interaction= 0.086 | | | | | | | | |
| Rheumatoid arthritis | |  |  |  |  |  |  |  |
| No | 57638 | 13043 |  | 57643 | 7091 | 2.09 | 2.03-2.15 | <0.001 |
| Yes | 148 | 33 |  | 143 | 13 | 2.68 | 1.41-5.1 | 0.003 |
| p for interaction= 0.443 | | | | | | | | |
| Ankylosing spondylitis | |  |  |  |  |  |  |  |
| No | 57749 | 13069 |  | 57752 | 7097 | 2.09 | 2.03-2.16 | <0.001 |
| Yes | 37 | 7 |  | 34 | 7 | 0.87 | 0.31-2.49 | 0.799 |
| p for interaction= 0.097 | | | | | | | | |
| Systemic lupus erythematosus | | |  |  |  |  |  |  |
| No | 57734 | 13062 |  | 57741 | 7094 | 2.09 | 2.03-2.16 | <0.001 |
| Yes | 52 | 14 |  | 45 | 10 | 1.27 | 0.56-2.87 | 0.561 |
| p for interaction= 0.233 | | | | | | | | |
| Sjogren’s syndrome | |  |  |  |  |  |  |  |
| No | 57731 | 13058 |  | 57725 | 7094 | 2.09 | 2.03-2.15 | <0.001 |
| Yes | 55 | 18 |  | 61 | 10 | 2.11 | 0.97-4.57 | 0.059 |
| p for interaction= 0.924 | | | | | | | | |
| Dyspepsia |  |  |  |  |  |  |  |  |
| No | 57171 | 12912 |  | 57220 | 7024 | 2.09 | 2.03-2.16 | <0.001 |
| Yes | 615 | 164 |  | 566 | 80 | 2.03 | 1.55-2.65 | <0.001 |
| p for interaction= 0.882 | | | | | | | | |
| GERD |  |  |  |  |  |  |  |  |
| No | 57641 | 13061 |  | 57659 | 7095 | 2.09 | 2.03-2.16 | <0.001 |
| Yes | 145 | 15 |  | 127 | 9 | 1.53 | 0.67-3.5 | 0.313 |
| p for interaction= 0.454 | | | | | | | | |
| Irritable bowel syndrome | | |  |  |  |  |  |  |
| No | 57465 | 12997 |  | 57505 | 7062 | 2.10 | 2.04-2.16 | <0.001 |
| Yes | 321 | 79 |  | 281 | 42 | 1.70 | 1.17-2.47 | 0.006 |
| p for interaction= 0.308 | | | | | | | | |
| Gastrointestinal tract cancer | | |  |  |  |  |  |  |
| No | 57297 | 13042 |  | 57326 | 7077 | 2.10 | 2.04-2.16 | <0.001 |
| Yes | 489 | 34 |  | 460 | 27 | 1.40 | 0.85-2.33 | 0.189 |
| p for interaction= 0.113 | | | | | | | | |
| Colonic polyp |  |  |  |  |  |  |  |  |
| No | 57720 | 13063 |  | 57733 | 7098 | 2.09 | 2.03-2.15 | <0.001 |
| Yes | 66 | 13 |  | 53 | 6 | 1.78 | 0.68-4.68 | 0.244 |
| p for interaction= 0.738 | | | | | | | | |
| Anxiety |  |  |  |  |  |  |  |  |
| No | 56802 | 12826 |  | 56855 | 6977 | 2.09 | 2.03-2.16 | <0.001 |
| Yes | 984 | 250 |  | 931 | 127 | 2.08 | 1.68-2.57 | <0.001 |
| p for interaction= 0.974 | | | | | | | | |
| Depression |  |  |  |  |  |  |  |  |
| No | 57202 | 12963 |  | 57259 | 7022 | 2.10 | 2.04-2.16 | <0.001 |
| Yes | 584 | 113 |  | 527 | 82 | 1.31 | 0.99-1.75 | 0.059 |
| p for interaction= 0.001 | | | | | | | | |
| Hypothyroidism | |  |  |  |  |  |  |  |
| No | 57664 | 13054 |  | 57676 | 7088 | 2.09 | 2.04-2.16 | <0.001 |
| Yes | 122 | 22 |  | 110 | 16 | 1.36 | 0.72-2.6 | 0.346 |
| p for interaction= 0.228 | | | | | | | | |
| Parkinson’s disease | |  |  |  |  |  |  |  |
| No | 57539 | 13060 |  | 57536 | 7091 | 2.10 | 2.04-2.16 | <0.001 |
| Yes | 247 | 16 |  | 250 | 13 | 1.14 | 0.55-2.36 | 0.733 |
| p for interaction= 0.104 | | | | | | | | |
| Spinal cord injury | |  |  |  |  |  |  |  |
| No | 57731 | 13068 |  | 57738 | 7102 | 2.09 | 2.03-2.15 | <0.001 |
| Yes | 55 | 8 |  | 48 | 2 | 3.91 | 0.83-18.46 | 0.085 |
| p for interaction= 0.467 | | | | | | | | |
