# Supplementary material for: Constipation might be associated with risk of allergic rhinitis: A nationwide population-based cohort study
Source: PLoS One. 2020 Oct 2;15(10):e0239723. doi: 10.1371/journal.pone.0239723 (PMC7531808; doi:10.1371/journal.pone.0239723)
Supplement: Attachment — Submitted filename: O-2020-002317 R2 Response letter 20200909.docx [file pone.0239723.s002.docx]

Tomohiko Ai, M.D., Ph.D.

Academic Editor

**Journal**: PLOS ONE

Dear Dr. Tomohiko Ai

On behalf of my coauthors, I thank the Editor/Reviewers for the comments and am grateful for the opportunity to revise our manuscript (ID: PONE-D-20-09268 R1) titled, “Constipation is associated with risk of allergic rhinitis: A nationwide population-based cohort study”, for consideration of publication as an Original Research in PLOS ONE. Your comments were highly insightful and enabled us to improve the quality of our document. In the following pages are our responses to each comment.

Revisions in the text are shown [yellow highlights]. We have addressed the comments point-by-point, and the corresponding changes have been made in track change in the manuscript, which you will find uploaded alongside this document. We hope that our revisions to the document combined with our accompanying responses will be sufficient to render our document suitable for publication in PLOS ONE.

We look forward to hearing from you.

Kind regards,

James Cheng-Chung Wei, MD, PhD

Institute of Medicine, Chung Shan Medical University

No. 110, Sec 1, Jianguo N. Road, Taichung, 40201, Taiwan

jccwei@gmail.com

**Responses to the Editor’s Comments:**

1. Also, the authors still overstate the results of statistical analyses (e.g., "Constipation was significantly associated with an increased risk of incidental AR"). It should be "might be associated."

**Response:**

Thank you for your comments and suggestions. We have toned down and modified these statements seen everywhere in the paper including the title.

For example, we have revised the wording in conclusion of abstract:

“Constipation might be associated with an increased risk of incidental AR.”

2. The authors should strongly emphasize the limitation of study (e.g., lack of laboratory data including cytokines and gene-expression changes, etc.).

**Response:**

Thank you for your comments and suggestions. We have added the wording in limitations of discussion section:

“Thirdly, relevant clinical information, such as laboratory data including cytokines and gene-expression changes, imaging findings, and fecal microbiota assessments were unavailable in the database and therefore could not be included in the analyses.”

We thank again from you for your best comments. Dear editor, your comments help us to promote quality of this manuscript and we appreciate your kind suggestions.

**Responses to the Reviewers’ Comments:**

**Manuscript ID:** **PONE-D-20-09268 R1**

**Manuscript title:** Constipation is associated with risk of allergic rhinitis: A nationwide population-based cohort study

**Journal: PLOS ONE**

Editor/Reviewer(s)' Comments to Author:

**Reviewer #1**: It is really interesting and well done work. This study after corrections is more interesting, sounds better and the data are presented clearer. However I have another comment (minor): I think the conclusion is slightly too restrictive, especially second sentence... Of course there were a great number of patients, good statistical tools but it was retrospective analysis and not meta analysis too. Therefore I propose be less restrictive in conclusion and use a term: it seems..."

Best regards

**Response:**

Thank you for your comments and suggestions.

We have changed the wording in conclusion as “It seems that physicians should keep a higher index of suspicion for AR in people with constipation.”

We thank you again for your best comments. Dear reviewer, your comments help us to promote quality of this manuscript and we appreciate your kind suggestions.

**Reviewer #2**: A propensity-matched analysis was conducted to show the association of constipation on time to allergic rhinitis (AR) development. After adjusting for relevant factors, patients with constipation had a 2.3 fold risk of AR compared to those without constipation.

Minor revisions:

1- Abstract: Methods: Replace the word “stratified” because the term causes confusion when it is not used based on its statistical definition. Consider using the term subgroup instead.

**Response:**

Thank you for your comments and suggestions. We have changed the wording as suggested in line 38 of abstract by using the term “subgroup”

2- Line 153: Provide a reference for the specific software procedure used to conduct the propensity-score matching.

**Response:**

Thank you for your comments and suggestions. We have attached the reference as suggested in line 154 and in the reference section.

Reference:

Austin PC. An Introduction to Propensity Score Methods for Reducing the Effects of Confounding in Observational Studies. Multivariate Behav Res. 2011;46(3):399-424.

3- Line 159: For the Kaplan-Meier analysis indicate the start time.

**Response:**

Thank you for your comments and suggestions. We have added the wording as suggested in line 162 “….was used to calculate the cumulative incidence of AR from the index date”.

4- In the statistical methods section, state the statistical testing methods used to generate the p-values in Table 1.

**Response:**

Thank you for your comments and suggestions. We have added in the statistical methods section and p-values in Table 1.

5- Line 216: Indicate the methods used to retain the adjustment variables of age, gender, comorbidities and medications in the model.

**Response:** Thank you for your comments and suggestions.

In general, from a statistical point of view, we could select the covariate p< 0.2 into multivariate analysis through univariate analysis. However, this might ignore the potential confounding factors in this work. On the other hand, from the perspective of study design and related research using the NHIRD dataset, as well as the comorbid conditions and medications suggested by Editor, all the covariates of age, gender, comorbidities and medications were adjusted in multivariate analysis in this study.

In addition to the baseline comorbidities used in our research, other more important variables for constipation and developing AR as editor’s suggestions were worth exploring. We have analyzed and adjusted a variety of comorbid conditions and medications in detail based on the reference (Nat Rev Dis Primers. 2017 Dec 14;3:17095) suggested by Editor’s comments in R1 revision. Finally, we also considered the most chronic gastrointestinal conditions, such as dyspepsia, GERD, IBS, colonic polyps, GI tract cancers. Psychiatric/Mood disorders such as anxiety and depression; autoimmune diseases including SLE, RA, AS, Sjogren’s syndrome; neurological diseases such as Parkinson’s disease, multiple sclerosis, spinal cord injury and hypothyroidism were also enrolled for analysis. In addition, we further considered various medications associated with constipation including antihistamines, corticosteroids, non-steroidal anti-inflammatory drugs, calcium channel blockers, diuretics, opioids, antidepressants, serotonin antagonists, anticonvulsants, antispasmodic, iron supplement, calcium supplement. All covariates were adjusted in multivariate analysis.

We have added the wording below table 2:

Multiple Cox proportional hazard regression was used to adjust for age, gender, comorbidities, and medications.

6- Table 2: For age, the multivariate Cox model should provide an overall p-value for comparing all the categories of age. If significant, a step-down test can be used to make pairwise comparisons. Indicate the overall p-value.

**Response:**

Thank you for your comments and suggestions. We have added the overall p-value of age in table 2.

7- Table 3:

(a) Provide a more descriptive title. See note #1 above.

**Response:**

We have revised the table 3 title as “Subgroup analysis of hazard ratios (95% CI) of AR for patients with and without constipation by age, gender, and comorbidities.”

(b) Table 3: Provide a description for each test of interaction by indicating the variables in the interaction term.

**Response:**

Thank you for your comments and suggestions. The interaction effect was to compare the hazard ratios between different subgroups. To make the description more clear, we had added a footnote in table 3 as ^†^p for difference of HR between subgroup and dropped the word “for interaction” in table 3.

And also added the description in results section:

The interaction effect was to compare the hazard ratios between different subgroups. In subgroup of non-hypertension, non-hyperlipidemia, non-chronic liver disease, and non-depression, constipation group had significant higher risk of allergic rhinitis”.

(c) Table 3: Clarify what the p-values represent. For instance, in those age <20 the log-rank p-value for comparing the time to allergic rhinitis between those with constipation and those without is <0.001. Is this interpretation accurate?

**Response:**

Thank you for your comments and suggestions. The p-value was from the Cox proportional hazard regression. This interpretation was constipation cohort had 2.09 fold risk of allergic rhinitis compared with non-constipation cohort in age <20. And the p value was <0.001.

(d) In general, if the interaction effect is significant, provide an interpretation of the results, but do not test main effects because the tests for main effects are uninteresting in light of significant interactions. If interaction effects are non-significant, drop the interaction effects from the model and test the main effects. Determining which

results to present when testing interactions is often a multi-step process.

**Response:**

Thank you for your comments and suggestions. The interaction effect was to compare the hazard ratios between different subgroups. We had added a footnote in table 3 as ^†^ p for difference of HR between subgroup and dropped the word “for interaction” in table 3.

8- Figure 2: Indicate the start time on the x-axis. “Years from xx.)

**Response:**

Thank you for your comments and suggestions. We have changed the x-axis as “Years from the index date”.

We thank you again for your best comments and suggestions. Dear reviewer, your comments help us to promote quality of this manuscript and we appreciate your kind suggestions.

**Reviewer #3**: This is a large epidemiological study using the National Health Insurance Research Database in Taiwan. Propensity score matching is used to analyze the association between constipation and allergic rhinitis, taking into account possible confounding. Despite the limitations of this study, I think that the results of the study suggest an association between gut microbiota and allergic rhinitis.

**Response:**

Thank you for your comments and suggestions.

Dear reviewer, I greatly appreciate your time and your kind comments again; your comments reinforced our manuscript and we did our best to respond to your comments carefully.
